# Interparental Conflict and Delinquency Among Chinese Adolescents: Parental Knowledge as a Mediator and Deviant Peer Affiliation as a Moderator

**DOI:** 10.3389/fpsyg.2020.01775

**Published:** 2020-08-20

**Authors:** Hong Lu, Quanfeng Chen, Chuyin Xie, Qiao Liang, Lanping Wang, Min Xie, Chengfu Yu, Jianping Wang

**Affiliations:** ^1^Center for Brain and Cognitive Sciences, School of Education, Guangzhou University, Guangzhou, China; ^2^School of Education, Research Center of Adolescent Psychology and Behavior, Guangzhou University, Guangzhou, China; ^3^School of Fine Arts and Design, Guangzhou University, Guangzhou, China; ^4^Longhu Middle School Affiliated to Guangdong University of Education, Shantou, China; ^5^School of Politics and Public Administration, Research Center of Education and Social Integration for the Guangdong-Hong Kong-Macao Greater Bay Area, South China Normal University, Guangzhou, China

**Keywords:** adolescent, interparental conflict, delinquency, parental knowledge, deviant peer affiliation

## Abstract

Interparental conflict has been found to positively affect adolescent delinquency; however, the underlying mechanism that explains this association remains unclear. This study investigated whether parental knowledge mediates the association between interparental conflict and adolescent delinquency, and whether this mediating process is moderated by deviant peer affiliation. To examine this, a total of 3,129 Chinese adolescents (47.27% boys, *Mean*_age_ = 14.94 years) completed a survey. Structural equation modeling indicated that the positive association between interparental conflict and adolescent delinquency is mediated by parental knowledge. Moreover, for adolescents with high deviant peer affiliation, interparental conflict was found to positively predict delinquency via parental knowledge; however, this indirect link was non-significant for adolescents with low deviant peer affiliation. These findings highlight the influence of parental knowledge and deviant peer affiliation on the association between interparental conflict and adolescent delinquency. This can provide guidance for the development of effective interventions that address the adverse effects of interparental conflict.

## Introduction

Adolescence is an important developmental period characterized by an increased risk of engaging in delinquent behaviors (Brunelle et al., 2012; Chiang et al., 2020). Delinquency is defined as various problem behaviors (such as stealing and alcohol use) that reflect negative reactions of adolescents to external environments (Wang et al., 2010). According to the China Justice Big Data Service Platform (2018), by 2017, the number of juvenile delinquents (adolescents who show problem behaviors that violate laws, such as stealing) in China exceeded 30,000, with junior high-school students representing a high-risk group. Extensive research has shown that adolescents who engage in delinquent behaviors are more inclined, when compared to those who do not engage in delinquent behaviors, to present adverse outcomes such as substance use, unemployment, and violent exposure (Becker et al., 2012; Carter, 2018; Burnside and Gaylord-Harden, 2019). Moreover, delinquency not only affects adolescents’ current health states and development but also negatively affects the stability and harmony of their social environments (Shek and Zhu, 2018). Thus, it is important to explore the external risk factors and possible mechanisms underlying delinquency, as this could contribute to the development of strategies for preventing the onset of delinquency and of interventions for adolescents who are already engaging in delinquent behaviors.

Among the many factors influencing adolescent delinquency, the role of parents has received considerable attention; in particular, research regarding the impact of interparental relationships on adolescent delinquency has a long history (Grych and Fincham, 1990; Davies and Cummings, 1994). Interparental conflict, which is an important negative occurrence that can develop in interparental relationships, refers to discord or physical aggression between parents regarding domestic issues (Grych and Fincham, 1990). Family, one of the most important elements of the social environment, provides a foundation for adolescents’ growth, with parents also representing a source of support, care, and trust (Li et al., 2016). However, parents who are involved in hostile and troubled relationships become more aggressive to their children and less sensitive to the children’s psychological needs (such as autonomy, relatedness, and competence; Sherrill et al., 2017; Zhang et al., 2017). There is considerable evidence that adolescents living in families with high interparental conflict are at an increased risk of developing serious mental-health problems and maladjustment (Schermerhorn et al., 2010; Ai et al., 2017; Harold and Sellers, 2018). Previous research has also demonstrated that interparental conflict is positively associated with adolescent delinquency (Vanassche et al., 2014; Liu et al., 2016; Xuan et al., 2018).

Although the relationship between interparental conflict and adolescent delinquency has been extensively investigated, there is no consensus regarding its mechanisms. In particular, few studies have examined the mediating role of parental knowledge and the moderating role of deviant peer affiliation in the case of Chinese youths. In an effort to bolster knowledge of the association between interparental conflict and delinquency among Chinese adolescents, this study aims to answer the following questions: (1) How is interparental conflict related to adolescent delinquency? (2) In what context does this association strengthen or attenuate?

### Parental Knowledge as a Mediator

Interparental conflict may have an influence on adolescent delinquency through reducing parental knowledge. Here, parental knowledge refers to parents’ extent of understanding regarding their adolescents’ whereabouts, companions, and other daily activities outside the home (Stattin and Kerr, 2000). Possessing a high level of knowledge regarding one’s adolescent child is a significant aspect of parenting (Bumpus and Hill, 2008). In fact, previous studies have found that parental knowledge is one of the most salient protective factors for preventing adolescents’ engagement in delinquency and misconduct (McAdams et al., 2014; Ornstein et al., 2018). According to the family systems theory, marital conflict may affect parent–adolescent interaction, which may, in turn, have an impact on adolescent adjustment (Cox and Paley, 2003). The concept inherent in this theory suggests that repeated exposure to interparental conflict hinders parents’ ability to maintain a high degree of knowledge regarding their adolescents, which increases the adolescents’ vulnerability to problem behaviors. In other words, parental knowledge, as a significant parent–adolescent interactive process, may be a mediator in the link between interparental conflict and delinquency. Although this has not yet been tested, some indirect evidence supports this theory regarding the mediation pathway.

First, interparental conflict appears to weaken parental knowledge. According to the “spillover effects” reported in the family systems theory, negativity aroused in one family sub-system is likely to spread to other family sub-systems; this includes the transmission of mood, affect, and behavior (Erel and Burman, 1995). Applying this in the context of the present study suggests that interparental conflict can “spill over” into the parent–child dyad (i.e., parental knowledge). Moreover, parents obtain information about their children through three primary methods: parental solicitation, child disclosure, and parental control. Parental solicitation refers to parents’ requests for information from adolescents or their friends; child disclosure refers to adolescents voluntarily sharing personal information with their parents; and parental control refers to active strategies, for example, setting rules concerning adolescent behavior (Stattin and Kerr, 2000). Notably, parental solicitation and parental control are parent-driven strategies for improving parental knowledge, while child disclosure is an adolescent-driven strategy.

Interparental conflict comprehensively affects parental knowledge through all of these three paths. On the one hand, as a result of frequent conflict, parents can become overwhelmed by their own problems and, thus, have less energy to invest in parenting efforts, such as caring for adolescents and soliciting information from them (Pereyra and Bean, 2017). Specifically, parents can become too exhausted to administer active behavioral control of adolescents, including setting expectations or rules for behaviors, setting boundaries on peer associations and activities, and even restricting the activities the adolescents can or cannot do without the parents’ consent. Thus, interparental conflict restricts parents’ ability to adopt parent-driven strategies for acquiring adolescents’ information. On the other hand, adolescents may spontaneously disclose personal information to parents if they perceive a presence of a positive, warm relationship in the family (Liu et al., 2019). However, perceiving intense and frequent interparental conflict signals an unstable and negative interparental relationship, which may result in adolescents developing a negative attitude regarding the parent–adolescent relationship. This could weaken the parent–adolescent bond and increase adolescents’ reluctance to eventually disclose information (Kerr et al., 2010).

Thus, when interparental conflict is present, parental knowledge regarding children’s daily lives is, in general, limited, with obstructions existing for both the parent-driven and adolescent-driven processes. In addition to the abovementioned research, several other studies have demonstrated that interparental conflict is associated with a negative parenting style and low parental knowledge (Buehler and Gerard, 2002; Brock and Kochanska, 2016; Xuan et al., 2018). For example, Brock and Kochanska found that interparental conflict is negatively associated with parent–child security (which signifies less child disclosure). Similarly, Fauchier and Margolin (2004) reported that parental conflict is a risk factor for parents’ withdrawal from a parenting role, which results in reduced parental knowledge.

Second, poor parental knowledge may increase adolescent delinquency. When parents have adequate parental knowledge, they can ascertain the activities their adolescents engage in during unsupervised leisure time, and can set rules and make timely interventions to prevent their children from engaging in delinquency (Ding et al., 2017). Conversely, poor parental knowledge contributes to parents becoming increasingly ineffective regarding providing guidance and leadership. This situation can result in adolescents obtaining excessive freedom and becoming prematurely autonomous, which can increase their opportunities to develop delinquent behaviors (Dishion et al., 2004; Fosco et al., 2012; Cutrín et al., 2017a). Several empirical studies have demonstrated that adequate parental knowledge may be associated with a lower risk of adolescents engaging in delinquent behaviors (Lahey et al., 2008; King et al., 2018; Scheuerman et al., 2019).

Although prior studies have examined the mediating role of parenting factors (i.e., the parent–child relationship) in the relationship between interparental conflict and juvenile delinquency (Gao et al., 2019), to our knowledge, few studies have explored the mediating role of parental knowledge in this regard. Therefore, the current study aims to extend the literature by examining the mediating effect of parental knowledge in this relationship. Based on existing literature and related theories, we propose the following hypothesis:

Hypothesis 1: Parental knowledge mediates the relationship between interparental conflict and adolescent delinquency.

### Deviant Peer Affiliation as a Moderator

Although interparental conflict may increase the risk of adolescent delinquency through the mediation of parental knowledge, adolescents who are exposed to interparental conflict do not experience equivalent levels of delinquency. This discrepancy may be the result of the influence of other associated contextual factors that modify the effect of interparental conflict on adolescent delinquency. According to the developmental contextualism theory proposed by Lerner (2001), individual development is affected not only by the respective influences of family and peer settings but also by dynamic interactions between different developmental environments. In the current study, we examine whether deviant peer affiliation moderates the link between interparental conflict and delinquency. Deviant peer affiliation is defined as association with peers who engage in deviant behaviors, such as cheating, fighting, and stealing (Rudolph et al., 2014). Adolescents are vulnerable to peer influence (Monahan et al., 2009; Jia et al., 2017), and extensive research has confirmed that adolescents who affiliate with deviant peers, when compared to adolescents who avoid such affiliation, are more likely to engage in problematic behaviors (Bao et al., 2015; Hinnant et al., 2016; Maheux et al., 2020). However, to our knowledge, the present study is the first to test whether deviant peer affiliation among Chinese youths plays a moderating role in the relationship between interparental conflict and delinquency. We chose to examine deviant peer affiliation as a moderator for the following reasons: (a) among adolescents, peers play a pivotal role in the development of socialization skills; (b) such analysis can help to clarify the interactive effect of family and peer context on adolescent delinquency; and (c) it is possible that deviant peer affiliation, as a contextual factor, can be addressed through interventions.

In accordance with this line of enquiry, the developmental contextualism theory suggests that deviant peer affiliation moderates the indirect relationship between interparental conflict and delinquency. More specifically, deviant friends not only can directly affect adolescents by representing various “non-mainstream” behavior models (i.e., smoking, cheating, and stealing) but also can modify the effect of other contextual factors, such as parental knowledge of delinquency, on adolescents (Wang et al., 2017; Tian et al., 2019). According to the stress-buffering model, the beneficial effect of parental knowledge is more pronounced when adolescents are exposed to high-risk environments (e.g., when they have a higher degree of deviant peer affiliation; Rueger et al., 2016). More specifically, as deviant peer affiliation increases, the protective effect of parental knowledge also gradually increases. Moreover, the negative effect of deviant peer affiliation can be buffered by parental knowledge. In other words, the negative relationship between parental knowledge and delinquency may be much stronger among adolescents who have high deviant peer affiliation. Conversely, for adolescents with less deviant peer affiliation, the protective effect of parental knowledge against delinquency is weaker. Empirical studies have provided evidence of a moderating effect of deviant peer affiliation in this relationship. For example, Wang et al. (2017) highlighted that the promoting effect of parent–child attachment on psychological resilience is weakened when adolescents have higher numbers of deviant peers. Similarly, using a large sample of 2,188 teenagers, Hou et al. (2017) found that deviant peer affiliation moderates the negative relationship between parental monitoring and problem behaviors; specifically, the link was stronger for adolescents with high deviant peer affiliation when compared to those with low deviant peer affiliation. Based on these previous findings and theories, we propose the following hypothesis:

Hypothesis 2: Deviant peer affiliation moderates the indirect association between interparental conflict and adolescent delinquency. More specifically, the indirect relationship between interparental conflict and adolescent delinquency via parental knowledge is stronger among adolescents with high levels of deviant peer affiliation and weaker among adolescents with low levels of deviant peer affiliation.

### The Present Study

**FIGURE 1:**
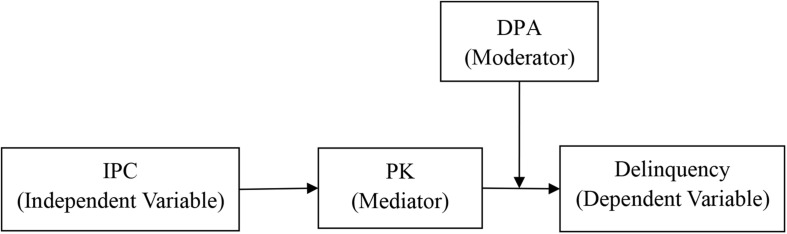
The proposed moderated mediation model. Parental knowledge mediates the association between interparental conflict and delinquency. Moreover, deviant peer affiliation moderates the indirect path between interparental conflict and delinquency. Gender and area were dummy-coded such that 1 = male, 0 = female. IPC, interparental conflict; DPA, deviant peer affiliation; PK, parental knowledge.

Guided by the family systems theory and the developmental contextualism theory, this study examines the impact of different contextual factors on adolescent delinquency. The overall purposes of this study are as follows: first, the study examines whether parental knowledge mediates the pathway between interparental conflict and delinquency; second, the study examines whether the mediating process between interparental conflict and delinquency via parental knowledge is moderated by deviant peer affiliation. These research questions describe a moderated mediation model that can address, among Chinese adolescents, both the mediation and the moderation mechanisms that exist in the relationship between interparental conflict and delinquency. Figure 1 illustrates the conceptual model.

## Materials and Methods

### Participants

Study participants were recruited from six middle schools in Guangdong Province, southern China, using stratified and random cluster sampling. The sample comprised a total of 3,129 adolescents (47.27% male), ranging in age from 10 to 19 years [*Mean*_age_ = 14.94, standard deviation (*SD*) = 1.97], giving an effective return rate of 94.82%. All participants were Han Chinese. Regarding the sample’s demographics, 64.95% of the participants’ fathers and 74.43% of their mothers had below high-school-level education; 65.90% came from rural areas, and 34.10% came from cities. Moreover, for 53.77% of the participants, their families had a monthly per-person income between ¥1,000 and ¥5,000 (the equivalent of $141–706). In 2019, the monthly per-person income for families in China was ¥3,530 (equivalent to approximately $498) for those in rural areas and ¥1,335 (approximately $189) for those in urban areas. Finally, 95.62% of the respondents had two-parent families, and 4.38% had stepfamilies. There were no significant differences between adolescents who came from two-parent families and those who came from stepfamilies with regard to ratings for interparental conflict and parental knowledge.

### Measures

#### Interparental Conflict

Interparental conflict was measured using the Conflict Properties Subscale of the Children’s Perception of Interparental Conflict Scale (Grych and Fincham, 1990). To complete this subscale, the participants were asked to answer 17 items that concerned their perceptions of interparental conflict in their families, including frequency, intensity, and resolution. All items were rated using a four-point scale (1 = “definitely disagree,” 4 = “completely agree”). For each participant, his/her average score for all 17 items was determined, with higher scores indicating higher levels of interparental conflict. The scale has shown good reliability in previous research (Chi and Xin, 2003; Liu et al., 2016). For the current study, the measure demonstrated outstanding reliability (α = 0.90).

#### Delinquency

The participants’ delinquency was assessed using the Chinese version of the Problem Behavior Scale (Wang et al., 2010). The participants were asked to report, using a five-point scale (1 = “never,” 5 = “five times or more”), the number of times they had engaged in various problem behaviors (e.g., alcohol use, robbery, blackmail/making threats, and truancy) during the last 6 months. For each participant, his/her average score for all items was determined, with higher scores representing greater delinquency. For the current study, the measure demonstrated good reliability (α = 0.77).

#### Parental Knowledge

Parental knowledge was assessed using a five-item version of the Parental Monitoring Questionnaire (Stattin and Kerr, 2000), which has previously been found to have good reliability for examinations of large Chinese samples (Cronbach’s alpha = 0.83; Jiang et al., 2016). The participants were asked to report, for the past 6 months, the extent of their parents’ knowledge regarding their activities during their leisure time, where they went after school, where they stayed when out at night, who they associated with, and how they spent their money. All five items were rated using a three-point scale (1 = “know little,” 3 = “know a lot”). For each participant, his/her average score for all five items was determined, with higher scores indicating higher parental knowledge. For the current study, the measure demonstrated good reliability (α = 0.77).

#### Deviant Peer Affiliation

Adolescents’ deviant peer affiliation was measured using the Chinese version of the Deviant Peer Affiliation Questionnaire (Zhu et al., 2015). The participants were asked to report the number of their friends who had shown deviant behaviors in the past 6 months (e.g., “How many of your friends have engaged in fights during the past 6 months?”). All items were assessed using a five-point scale (1 = “none,” 5 = “almost all”). For each participant, his/her average score for all 12 items was determined, with higher scores showing higher levels of deviant peer affiliation. For the current study, the measure demonstrated excellent reliability (α = 0.88).

#### Covariates

Given that adolescent gender, age, and impulsivity have been found to be correlated with delinquency (Chen and Jacobson, 2013; McMillian et al., 2018), we controlled for these variables in our statistical analyses. Impulsivity was measured using a short version of the Negative Urgency–Premeditation–Perseverance–Sensation seeking–Positive Urgency (UPPS-P) Scale (Cyders et al., 2014). For this scale, the participants gave their responses using a four-point scale ranging from 1 (“strongly disagree”) to 4 (“strongly agree”). Given the relatively high correlations among these five dimensions (i.e., negative urgency, premeditation, perseverance, sensation seeking, and positive urgency), a composite score was created for each participant by determining his/her overall average score for all 20 items; higher scores indicated higher levels of impulsivity. Many empirical studies have used the UPPS-P Scale to measure adolescent impulsivity and have calculated the mean scores for all items when performing their analysis (Liu et al., 2016; Zhu et al., 2016). For this study, the UPPS-P Scale demonstrated excellent reliability (Cronbach’s alpha = 0.82).

### Procedure

This research was approved by the Ethics Review Committee of the School of Education, Guangzhou University. Informed consent was obtained from the teachers, parents, and students before the assessment commenced. Trained psychology graduate students conducted the investigation in the participants’ classrooms. The participants were asked to complete the self-report questionnaires in accordance with the standardized instructions. To encourage truthful answers, participants were informed that their responses would be kept confidential. The participants completed the questionnaires in approximately 30 min, and the questionnaires were collected immediately thereafter. Before the survey, participants received a pen as a reward for their participation.

### Statistical Analysis

SPSS 20.0 was used to derive descriptive statistics for all variables. To test the mediation and moderation effects, we conducted structural equation modeling using maximum likelihood estimation and bootstrapping with 1,000 replicates. The statistical analysis software used was Mplus 7.1 (Muthén and Muthén, (1998–2012)).

## Results

### Preliminary Analyses

The means, standard deviations, and correlation coefficients for all variables examined in the current study are displayed in Table 1. The results showed that interparental conflict and deviant peer affiliation were both negatively correlated to parental knowledge (*r* = −0.26, *p* < 0.01; *r* = −0.17, *p* < 0.01) and were both positively correlated to delinquency (*r* = 0.11, *p* < 0.01; *r* = 0.35, *p* < 0.01). Moreover, parental knowledge scores were negatively correlated to delinquency (*r* = −0.24, *p* < 0.01).

**TABLE 1:** Descriptive statistics and correlations for all variables.

Variables	1	2	3	4	5	6	7
1. Gender	1.00						
2. Age	–0.03	1.00					
3. Impulsivity	0.00	0.19**	1.00				
4. IPC	−0.04*	0.20**	0.29**	1.00			
5. DPA	0.12**	−0.06**	0.20**	0.11**	1.00		
6. PK	−0.13**	−0.28**	−0.30**	−0.26**	−0.17**	1.00	
7. Delinquency	0.15**	0.13**	0.19**	0.11**	0.35**	−0.24**	1.00
*Mean*	0.47	14.94	2.29	2.14	1.38	2.37	1.06
*SD*	0.50	1.97	0.37	0.63	0.64	0.49	1.06

*Gender and area were dummy-coded such that 1 = male, 0 = female. IPC, interparental conflict; DPA, deviant peer affiliation; PK, parental knowledge. ^∗^p < 0.05, ^∗∗^p < 0.01.*

### Testing for the Mediation Effect of Parental Knowledge

**FIGURE 2:**
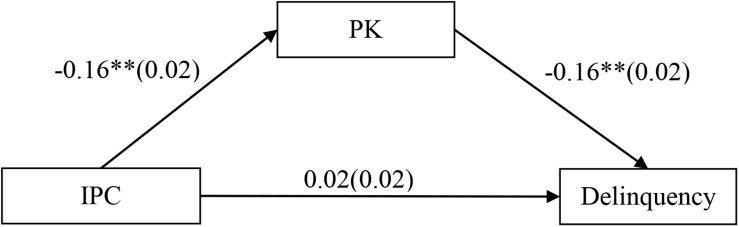
Model of the mediating role of parental knowledge in the relationship between interparental conflict and delinquency. Values (those that are not enclosed in parentheses) are unstandardized coefficients; the standard error is enclosed in parentheses. Paths between gender, age, impulsivity, and each of the variables in the model are not displayed. Of those paths, the following were significant: gender (*b* = −0.28, *SE* = 0.03, *t* = −8.64**), age (*b* = −0.21, *SE* = 0.02, *t* = −12.76**), and impulsivity (*b* = −0.21, *SE* = 0.02, *t* = −12.33**) to parental knowledge; and gender (*b* = 0.26, *SE* = 0.03, *t* = 7.54**), age (*b* = 0.06, *SE* = 0.02, *t* = 3.54**), and impulsivity (*b* = 0.12, *SE* = 0.02, *t* = 6.63**) to delinquency. **p < 0.01.

First, this study tested the direct effect (total effect “c”) between interparental conflict and delinquency. After controlling for gender, age, and impulsivity, it was found that interparental conflict had a significant direct effect on delinquency (*b* = 0.05, *SE* = 0.02, *t* = 2.74, *p* < 0.01). Next, we tested the mediation model, and the mediation model presented in Figure 2 showed an excellent fit to the data: χ^2^/*df* = 1.81, comparative fit index (CFI) = 1.00, root mean square error of approximation (RMSEA) = 0.016. Interparental conflict negatively predicted parental knowledge (*b* = −0.16, *SE* = 0.02, *t* = −9.34, *p* < 0.01), and parental knowledge negatively predicted delinquency (*b* = −0.16, *SE* = 0.02, *t* = −8.42, *p* < 0.01). However, the residual effect of interparental conflict on delinquency was non-significant (*b* = 0.02, *SE* = 0.02, *t* = 1.35, *p* > 0.05). Moreover, the bootstrapping analyses indicated that parental knowledge partially mediated the relationship between interparental conflict and adolescent delinquency (indirect effect = 0.0254, *SE* = 0.0049, 95% confidence interval [CI] [0.0167, 0.0362]).

### Testing for Moderated Mediation

**FIGURE 3:**
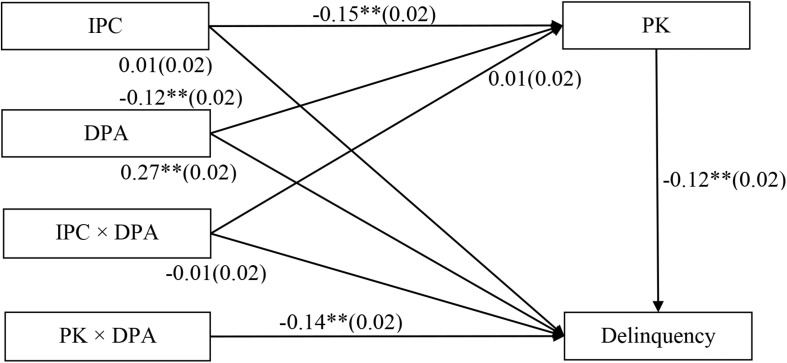
Model of the moderating role of deviant peer affiliation on the indirect relationship between interparental conflict and delinquency. Values (those that are not enclosed in parentheses) are unstandardized coefficients; the standard error is enclosed in parentheses. Paths between gender, age, father–adolescent relationship, mother–adolescent relationship, impulsivity, and each of the variables in the model are not displayed. Of those paths, the following were significant: gender (*b* = −0.25, *SE* = 0.03, *t* = −7.83**), age (*b* = −0.23, *SE* = 0.02, *t* = −13.55**), and impulsivity (*b* = −0.19, *SE* = 0.02, *t* = −10.79**) to parental knowledge; and gender (*b* = 0.21, *SE* = 0.03, *t* = 6.45**), age (*b* = 0.11, *SE* = 0.02, *t* = 6.13**), and impulsivity (*b* = 0.07, *SE* = 0.02, *t* = 3.79**) to delinquency. **p < 0.01.

**FIGURE 4:**
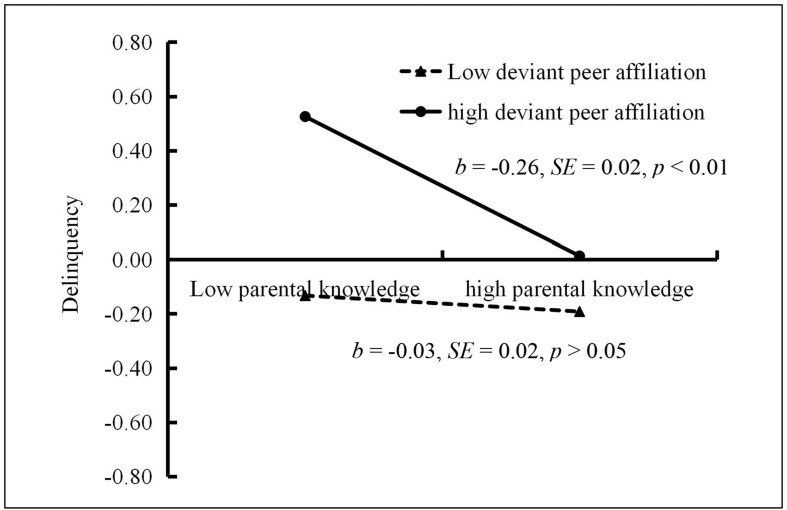
Association between parental knowledge and delinquency at higher and lower levels of deviant peer affiliation.

The moderated mediation model presented in Figure 3 showed a good fit to the data: χ^2^/*df* = 3.40, CFI = 0.96, RMSEA = 0.067. The bias-corrected percentile bootstrap results indicated that the indirect effect of interparental conflict on adolescent delinquency through parental knowledge was moderated by deviant peer affiliation. Specifically, deviant peer affiliation moderated the association between parental knowledge and delinquency (*b* = −0.14, *SE* = 0.02, *t* = −8.93, *p* < 0.01). We then conducted a simple slopes test, and as depicted in Figure 4, parental knowledge was found to be significantly associated with delinquency among adolescents with higher deviant peer affiliation (one *SD* below the mean; *b* = −0.26, *SE* = 0.02, *t* = −10.83, *p* < 0.01). However, this link between parental knowledge and delinquency was not significant among adolescents with lower deviant peer affiliation (one *SD* above the mean; *b* = −0.03, *SE* = 0.02, *t* = −1.45, *p* > 0.05). Moreover, deviant peer affiliation had a significant negative association with parental knowledge (*b* = −0.15, *SE* = 0.02, *t* = −8.53, *p* < 0.01) and a significant positive relationship with delinquency (*b* = 0.28, *SE* = 0.02, *t* = 15.97, *p* < 0.01). However, the interaction between interparental conflict and deviant peer affiliation with regard to predicting parental knowledge (*b* = 0.02, *SE* = 0.02, *t* = 1.06, *p* > 0.05) and delinquency (*b* = −0.01, *SE* = 0.02, *t* = −0.46, *p* > 0.05) was not significant.

Moreover, the indirect link between interparental conflict and delinquency via parental knowledge was significant for adolescents with higher deviant peer affiliation (indirect effect = 0.0364, *SE* = 0.0082, 95% CI [0.0235, 0.0563]). However, this indirect link was non-significant for those with lower deviant peer affiliation (indirect effect = 0.0046, *SE* = 0.0046, 95% CI [−0.0023, 0.0158]). Therefore, the mediating effect of parental knowledge in the relationship between interparental conflict and adolescent delinquency was moderated by deviant peer affiliation.

## Discussion

This study sought to examine the relationship between interparental conflict and delinquency among Chinese adolescents, with the inclusion of parental knowledge and deviant peer affiliation in the model. Consistent with hypothesis 1, we found that parental knowledge is a mediator in this association. This finding is also consistent with the family systems theory (Cox and Paley, 2003), which posits that parental knowledge, as a process of social interaction between parents and adolescents, is an underlying mechanism that helps explain why interparental conflict is linked with delinquency. As a familial stressor, interparental conflict can reduce parental knowledge, causing parents to be less informed about their adolescents, consequently resulting in adolescents being less likely to constraint their delinquent behaviors.

First, interparental conflict results in parents’ withdrawal from their adolescents’ lives, leading parents to spend less time engaging in family activities, pay less attention to their children, and provide less active supervision (Cox et al., 2001). Hence, such parents do not actively solicit information from their adolescents, which also prevents adolescents from revealing daily information about themselves to their parents (Tilton-Weaver et al., 2010). Moreover, the optimal familial atmosphere of understanding, trust, and communication is negatively affected by interparental conflict, which triggers a sense of emotional insecurity and further deters adolescent openness (Lahey et al., 2008; Cutrín et al., 2017b). Taken together, these findings indicate that adolescents who are exposed to interparental conflict are inclined to report low levels of parental knowledge.

Second, parental knowledge negatively predicts adolescent delinquency. Adequate parental knowledge leads to adolescents perceiving greater attachment, belonging, and care in interactions with their parents, while also perceiving that their parents value their relationship. Thus, the adolescents may reduce their engagement in delinquent behaviors lest it negatively impact their parents and the parent–adolescent relationship (Branstetter et al., 2009). Conversely, adolescents who perceive poor parental knowledge usually have a weak bond with their parents, which leads them to become less likely to empathize with their parents’ feelings and to feel guilty when they engage in delinquent behaviors. As a result, such adolescents have an increased risk of turning toward delinquency in terms of their developmental trajectory.

Consistent with hypothesis 2, the results also showed that deviant peer affiliation moderates the indirect link between interparental conflict and adolescent delinquency via parental knowledge. This result advances earlier research findings regarding Chinese youths by revealing the relationship between interparental conflict and adolescent delinquency in a moderated mediation model. More specifically, there is a significant interaction between parental knowledge and high deviant peer affiliation in terms of the negative prediction of delinquency; however, this prediction is not significant for low deviant peer affiliation. This finding is consistent with developmental contextualism theory (Lerner, 2001), which proposes that parents and peers have an interactive effect on adolescent delinquency. This finding also accords with the stress-buffering model (Rueger et al., 2016), which proposes that the negative relationship between parental knowledge and delinquency is much stronger among adolescents in high-risk environments, such as those with deviant peer affiliation. A possible explanation for this is that adolescents can acquire social support and a sense of belongingness from peers, even peers who engage in deviant behavior. Thus, when associating with deviant peers, adolescents who have weak relationships with their parents may try to integrate into the deviant groups by conforming to the group norms, as such integration might compensate for the feelings of insecurity they experience as a result of their relationships with their parents. Additionally, adolescents who are exposed to multiple risk contexts lack an “arena of comfort” in their lives (Simmons et al., 1987); as a consequence, they are likely to experience considerable discomfort with themselves and the world around them and to seek compensation by exhibiting delinquent behaviors. However, adolescents with a low level of deviant peer affiliation lack negative “role models” and peer pressure to exhibit delinquent behavior; thus, even if parents do not set rules or monitor their whereabouts, their likelihood of exhibiting delinquency is lower than that of adolescents with more deviant peers (Bandura and Walters, 1963). This finding also suggests that, compared with the positive effect of adequate parental knowledge, the negative effect of deviant peer affiliation on delinquency is much more powerful.

This study examined moderating effects of deviant peer affiliation that have previously been neglected in research in this area. The moderated mediation model formed in this study provides greater predictive power than the mediation model alone, increasing understanding of the development of delinquent behaviors among Chinese adolescents. Our research indicates that interparental conflict can affect adolescent delinquency by reducing parental knowledge. Moreover, the indirect association between interparental conflict and adolescent delinquency via parental knowledge was found to differ across adolescents; researchers who are interested in the impact of interparental conflict and adolescent delinquency and the associated variations should seek to identify the influence of adolescents’ companions in this regard.

This study has several limitations that must be acknowledged. First, this study featured a cross-sectional design, meaning it was not possible to make causal inferences. Second, this study principally relied on self-report measures and adolescent respondents. Although previous research has indicated that adolescents can provide valuable and accurate information regarding interparental conflict, parental knowledge, and delinquency (Dotterer and Wehrspann, 2016; Fan et al., 2018), caution should be exercised regarding the possible impacts of shared method variance and social desirability on the data. Further research should utilize additional respondents (e.g., multiple informants, including peers, teachers, and parents), which may capture more credible information and reduce the shared method variance. Third, our research only focused on general delinquency among adolescents. Further research should test the moderated mediation model for different forms of delinquent behaviors. Finally, the current findings are limited to adolescents from Guangdong Province, China. Further studies should explore whether this moderated mediation model accurately describes the development of delinquency among adolescents from other countries and regions.

Despite these limitations, this study has important implications regarding the prevention of delinquency among Chinese youths. First, this study highlights the association between interparental conflict and adolescent delinquency. Minimizing interparental conflict and creating a warm and accepting atmosphere for adolescents may be an effective method of reducing adolescents’ engagement in delinquent behaviors. Second, our findings reveal that parental knowledge plays an important role in linking interparental conflict and adolescent delinquency. Parents’ monitoring of their adolescents may help to prevent delinquency. Third, given that deviant peer affiliation serves as a moderator of the indirect association between interparental conflict and delinquency among adolescents, it is of great importance to identify and focus on adolescents who associate with deviant peers; practitioners should develop targeted interventions for such adolescents. Finally, our moderated mediation model suggests that adolescent behavior can be influenced by complex interactions with environmental factors such as family and peers. Therefore, to develop interventions for delinquency, risk factors relating to both parents and peers should be considered.

## Data Availability Statement

The datasets generated for this study are available on request to the corresponding author.

## Ethics Statement

The studies involving human participants were reviewed and approved by the Ethics Review Committee of School of Education, Guangzhou University. Written informed consent to participate in this study was provided by the participants’ legal guardian/next of kin.

## Author Contributions

HL, QC, CY, and JW conceived and designed the research, performed the research, and wrote the manuscript. CY and JW analyzed the data. HL, QC, CX, QL, LW, MX, CY, and JW revised the article critically for important intellectual content, commented on, and approved the final manuscript. All authors contributed to the article and approved the submitted version.

## Conflict of Interest

The authors declare that the research was conducted in the absence of any commercial or financial relationships that could be construed as a potential conflict of interest.
